# Supplementary material for: Adsorption of rare earth elements in regolith-hosted clay deposits
Source: Nat Commun. 2020 Sep 1;11:4386. doi: 10.1038/s41467-020-17801-5 (PMC7463018; doi:10.1038/s41467-020-17801-5)
Supplement: Supplementary file 1 — Supplementary Information [file 41467_2020_17801_MOESM1_ESM.pdf]

Supplementary Information

Adsorption of Rare Earth Elements in Regolith-hosted Clay Deposits

Anouk M. Borst[1*], Martin P. Smith[2], Adrian A. Finch[1], Guillaume Estrade[3], Cristina Villanova-de-Benavent[2], Peter Nason[2], Eva Marquis[2], Nicola J. Horsburgh[1], Kathryn M. Goodenough[4], Cheng Xu[5,6], Jindrich Kynicky[7,8], Kalotina Geraki[9]

[1] School of Earth and Environmental Sciences, University of St Andrews, St Andrews, KY16 9AL, UK

[2] School of Environment and Technology, University of Brighton, Brighton, BN2 4GJ, UK

[3] GET, University of Toulouse, CNRS, IRD, UPS, Toulouse, France

[4] British Geological Survey, The Lyell Centre, Research Avenue South, Edinburgh, EH14 4AP, UK

[5] College of Earth Sciences, Guilin University of Technology, Guilin 541006, China

[6] School of Earth and Space Sciences, Peking University, Beijing 100871, China

[7] Department of Geology and Pedology, Mendel University, Zemedelska 1, CZ-61300, Brno, Czech Republic

[8] BIC Brno Spol. s.r.o., Technology Innovation Transfer Chamber, Purkyňova 648/125, CZ-61200, Brno, Czech Republic

[9] Diamond Light Source, Physical Science, Harwell Science Campus, Didcot, OX11 0DE, UK

*Corresponding author:

amb43@st-andrews.ac.uk, or anoukborst@gmail.com

https://orcid.org/0000-0003-0775-1491

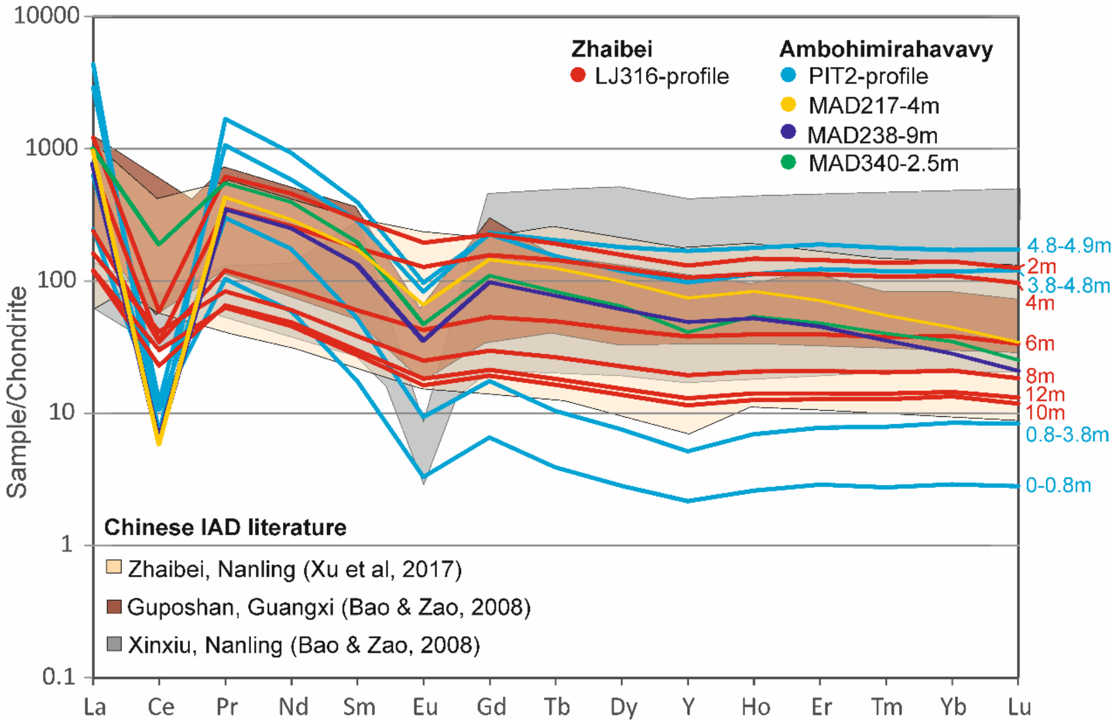

Supplementary Figure 1

Chondrite normalised profiles of the exchangeable REE fractions leached with an ammonium sulphate solution (0.5 M $(NH_4)_2SO_4$ adjusted at pH 4 with H_2SO_4) from the Zhaibei laterite profile (LJ316 – samples from 2 to 12 m) and the Malagasy laterite samples (PIT2 – samples from 0 to 4.9 m, MAD217, MAD340 and MAD238). Source data provided in Supplementary Table 1, for full dataset of the Malagasy samples see Estrade et al. 2019[1]. Also shown for comparison are literature whole rock data from Zhaibei[2] and other Chinese heavy REE-rich ion adsorption deposits (IAD) from Xinxiu, Longnan County and Guposhan, Guangxi County, China[3]. Samples selected for XAS were those with the highest exchangeable REE concentrations (also Supplementary Figure 2). Chondrite values from McDonough and Sun[4].

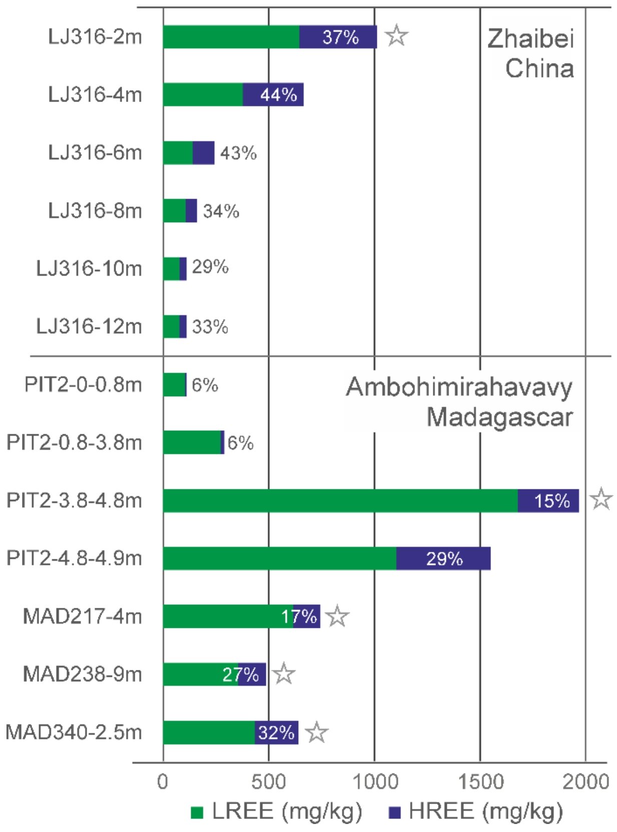

Supplementary Figure 2

Exchangeable light REE (La-Sm, green) and heavy REE (Eu-Lu, Y, blue) fractions from ammonium sulphate ($(NH_4)_2SO_4$) leaching of regolith samples from Zhaibei, China, and Ambohimirahavavy, Madagascar. Numbers indicate the relative proportions of heavy REE in percentages. Stars indicate the samples selected for X-ray Absorption Spectroscopy analyses. Rare Earth Element leaching data are provided in Supplementary Table 1.

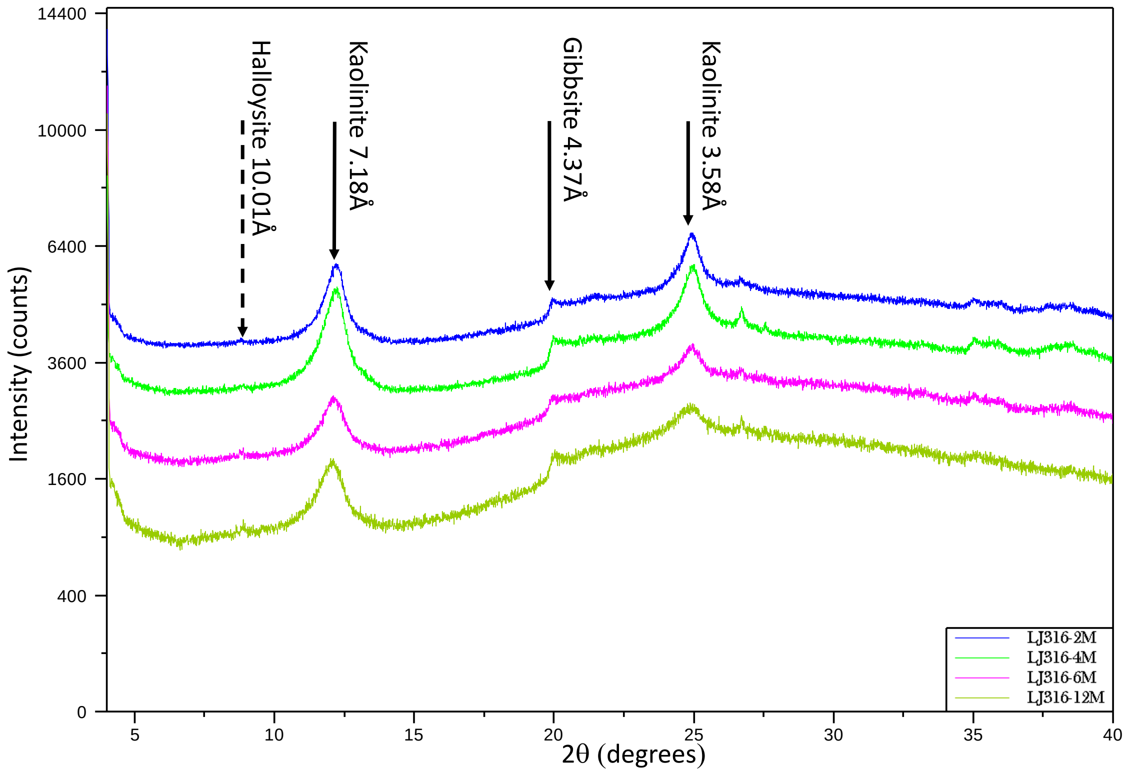

Supplementary Figure 3

X-ray diffractogram of oriented clay fraction from the Zhaibei LJ316 profile samples, showing the dominance of kaolinite, characterised by two prominent peaks at 7.18 Å and 3.58 Å (001 and 002 basal reflections, respectively), and minor gibbsite (at 4.37 Å) throughout the profile. The broadness of the kaolinite peaks could reflect high structural disorder or small particle size of kaolinite (Supplementary Figure 4). LJ316-2m (blue) was used for our study. The Zhaibei samples are distinct from the Malagasy samples in that halloysite-10 Å is largely absent, although very minor traces of a 10 Å peak (001 basal reflection) may be recognised in the deepest samples (6 and 12m). XRD data from the Madagascar samples measured in this study is available in Estrade et al, 2019[1] (Fig 5 and Appendix A therein). Although XRD did not detect halloysite-10 Å, the presence of halloysite-7 Å (through dehydration of halloysite-10 Å) cannot be ruled out and may contribute to the width of the 7 Å kaolinite peak.

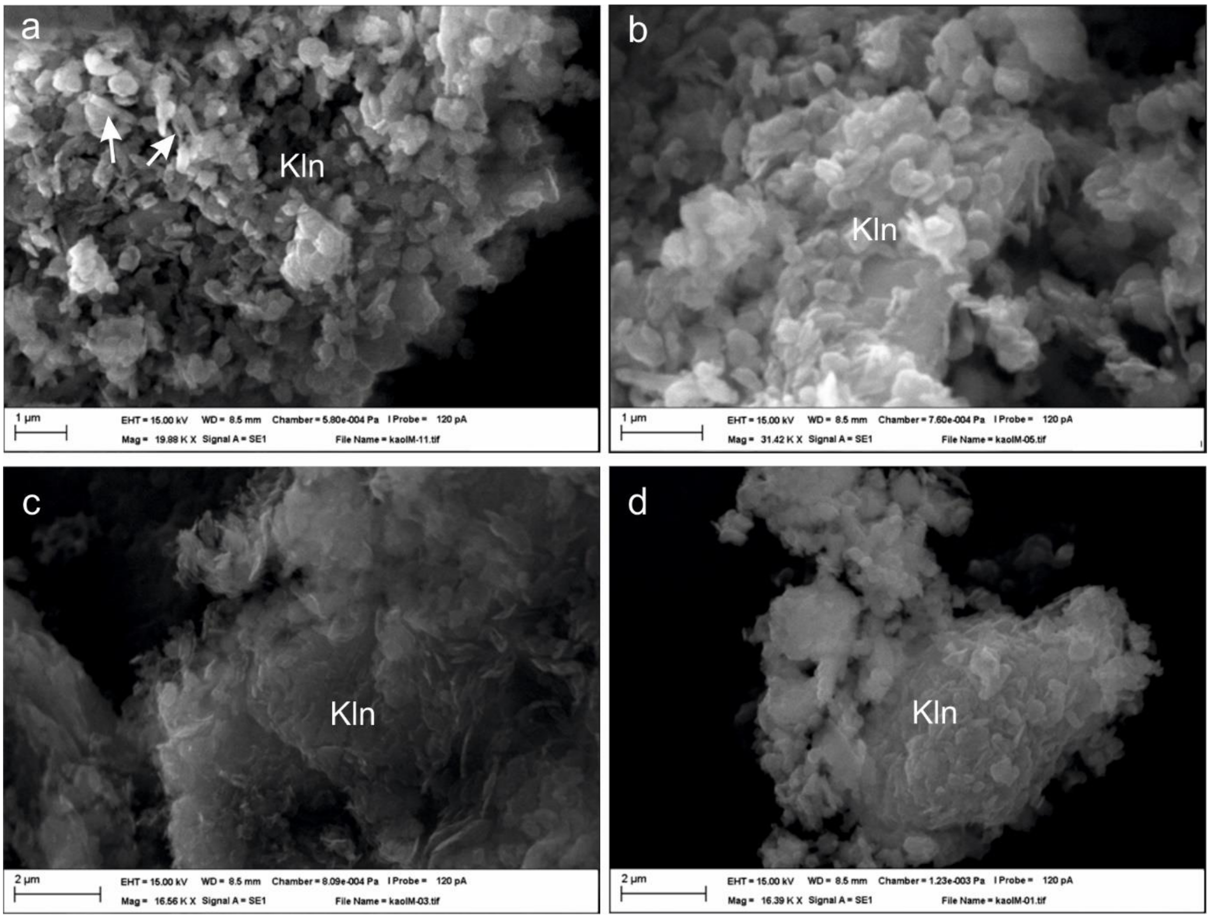

Supplementary Figure 4

Backscatter electron images obtained from the Scanning Electron Microprobe showing separated clay fractions from regolith samples of the Ambohimirahavavy complex, Madagascar, processed for isotopic analyses. The images demonstrate a dominant morphology of sub-micrometer sized subrounded to pseudohexagonal plates and thin stacks of microcrystalline kaolinite. White arrows point to conceivable tubular morphologies that might represent halloysite (10 Å or 7 Å), although these could also represent the edges of kaolinite stacks.

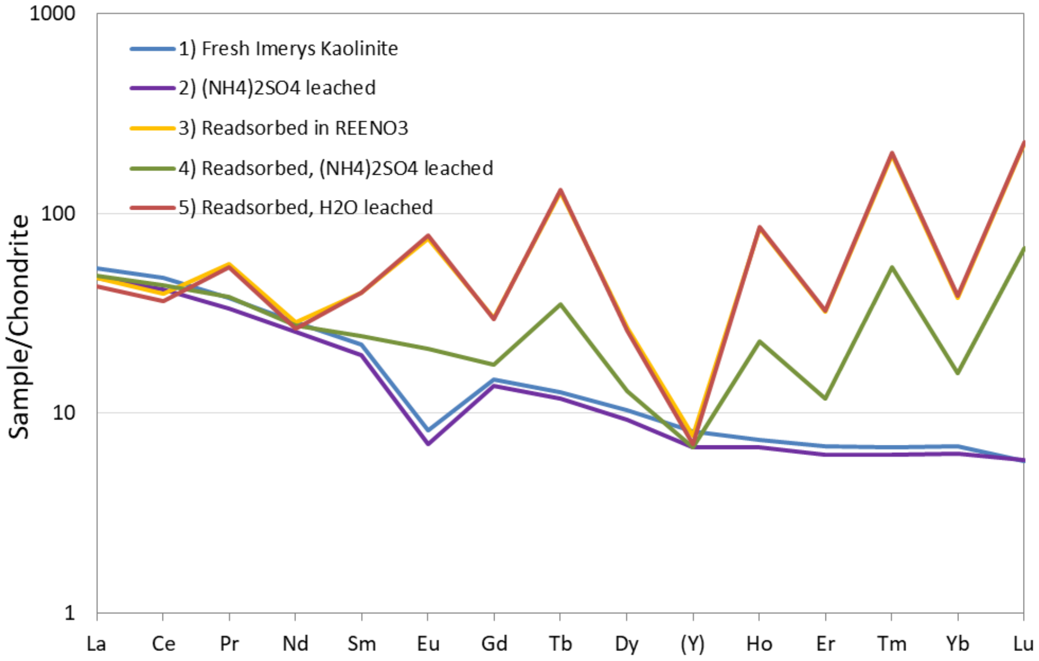

Supplementary Figure 5

Chondrite-normalised REE concentrations of prepared IMERYS kaolinite standards. The agreement between the bulk analyses of the fresh untreated IMERYS kaolinite (1) and the same material following ammonium sulphate ($(NH_4)_2SO_4$) leaching (2) indicates REE initially present are bound in the mineral structure. REE doped kaolinite (3) was prepared from ~30 ppm $REENO_3$ solution (based on Agilent REE standard) in the presence of 0.1M $NaNO_3$ with IMERYS Light kaolinite (1). The comparison between re-adsorbed kaolinite before (3) and after ammonium sulphate leaching (4) indicate that reversible adsorption of the REE has been achieved. Water leaching of the re-adsorbed kaolinite (5) does not show changes in concentrations, indicating REE are sorbed to the kaolinite. Chondrite values from McDonough and Sun [4]. REE concentration data for each of the IMERYS kaolinite leaching experiment steps, along with measured standards, are provided in Supplementary Table 2.

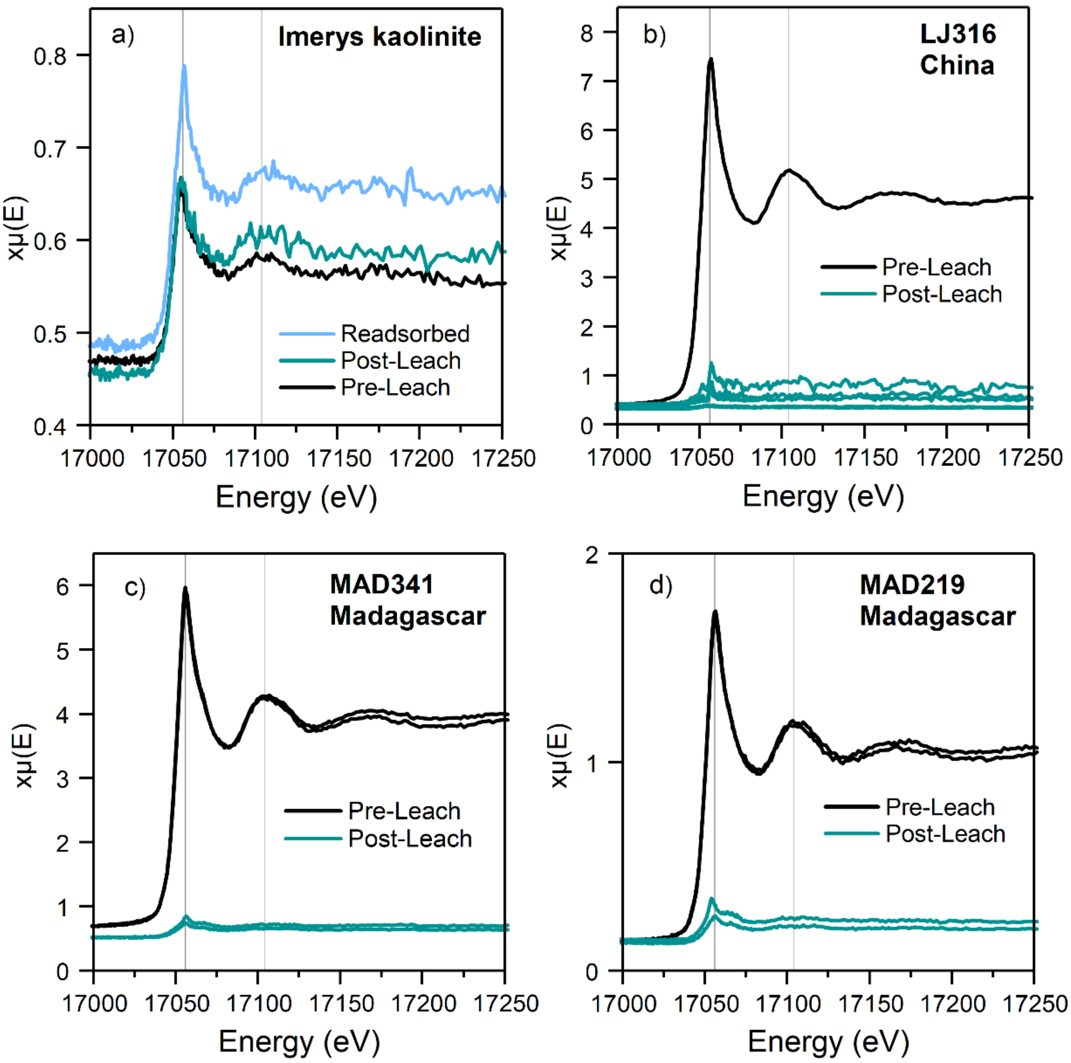

Supplementary Figure 6

Raw un-normalised Y K-edge XANES spectra for (a) the prepared IMERYS kaolinite standards, and the processed bulk powders derived from (b) the Chinese and (c,d) Malagasy laterite samples. XANES were measured on untreated material, and on samples splits post a multistage leaching procedure. The IMERYS kaolinite standard was also re-adsorbed with Y in a Y-bearing solution (Supplementary Figure 5). Differences in the height of the absorption edges between the pre-leach and post-leach demonstrate high Y concentrations in the pre-leach materials. The close comparison between the pre-leach bulk powder XANES and the *in situ* clay-hosted XANES spectra (Figure 4, main text) indicates that the kaolinite-hosted structural state of Y dominates the REE budget in both Chinese and Malagasy laterite samples. Data shown in the figure are provided in Supplementary Data 2.

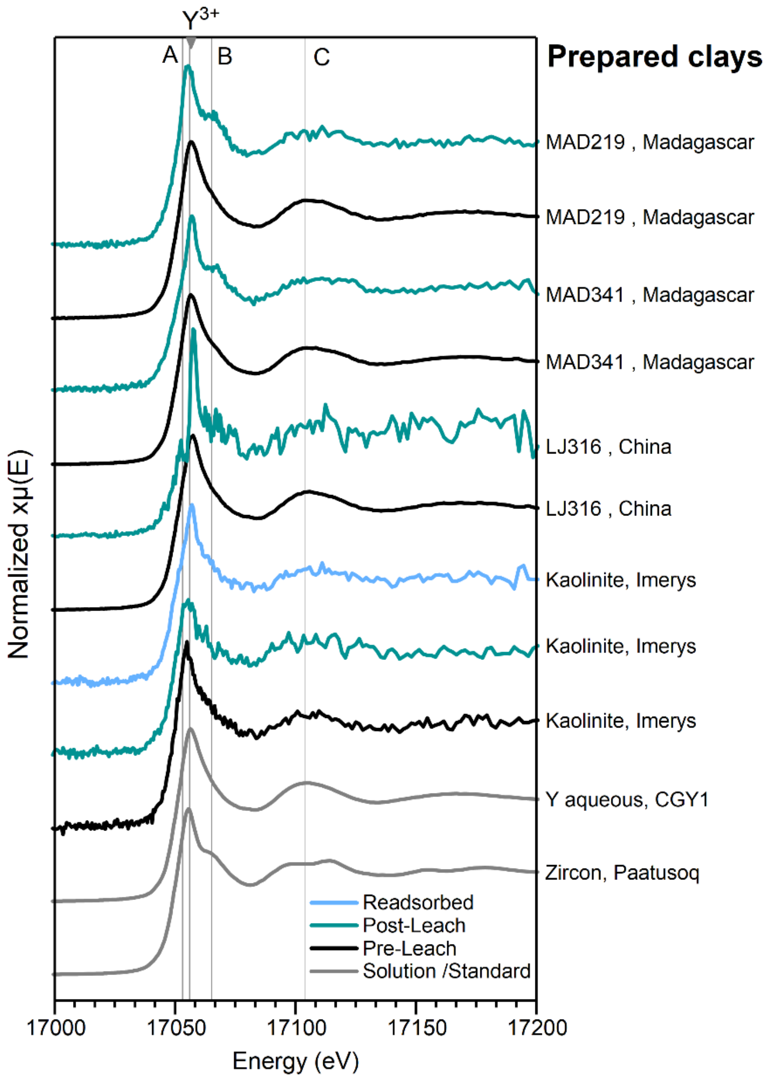

Supplementary Figure 7

Normalised Y K-edge XANES for the prepared IMERYS kaolinite standards and processed bulk powders of the Chinese and Malagasy regolith samples. XANES spectra for Y in solution and zircon are shown for comparison. Normalised spectra for the post-leach powders demonstrate a minor peak at 17064 eV (Feature B), which resembles spectral features of the high-point symmetry standards, such as zircon (Features A, B, C as in Figure 4 in main text). This suggests the non-leachable REE in the soils are hosted in higher-symmetry structural sites, either more strongly bound as inner-sphere complexes adsorbed to kaolinite, other clays and Fe-Mn oxyhydroxides, or as nanoscopic residual mineral phases. Details of the materials are provided in Supplementary Data 1, and the XANES data shown in the figure are provided in Supplementary Data 2.

Supplementary Table 1

Exchangeable Rare Earth Element concentrations by ammonium sulphate ($(NH_4)_2SO_4$) leaching of the studied samples

**Locality**	**Zhaibei**						**Ambohimirahavavy**						
**Country**	**China**						**Madagascar**						
**Sample**	**LJ316**						**PIT2 (BTPIT)**				**MAD217**	**MAD238**	**MAD340**
**Depth interval (m)**	**2**	**4**	**6**	**8**	**10**	**12**	**0-0.8**	**0.8-3.8**	**3.8-4.8**	**4.8-4.9**	**4**	**9**	**2.5**
in mg kg^{-1} (ppm)													
**La**	289.2	170.4	56.6	38.2	28.4	28.1	58.1	148.6	1031.2	685.4	238.7	183.3	230.2
**Ce**	36.2	21.6	21.0	25.4	18.3	14.0	6.5	6.5	4.2	7.4	115.3	3.8	3.5
**Pr**	56.9	32.7	11.1	7.7	5.8	6.1	9.6	27.9	156.0	98.7	50.8	32.3	40.0
**Nd**	208.7	118.4	39.6	27.5	20.7	22.1	27.0	79.8	425.0	265.8	179.9	113.9	132.2
**Sm**	43.2	26.2	8.9	5.6	4.1	4.4	2.6	7.7	58.5	42.4	28.9	19.6	25.5
**Eu**	11.0	7.2	2.4	1.4	0.9	1.0	0.2	0.5	5.5	4.7	2.6	2.0	3.7
**Gd**	44.7	31.2	10.6	5.9	3.8	4.2	1.3	3.5	44.8	45.9	21.7	19.4	28.8
**Tb**	6.9	5.2	1.8	1.0	0.6	0.7	0.1	0.4	5.5	7.4	3.0	2.8	4.5
**Dy**	38.8	30.5	10.5	5.6	3.4	3.8	0.7	1.8	29.3	44.3	15.8	14.9	24.3
**Y**	205.1	166.6	59.8	30.5	18.0	20.4	3.4	8.1	153.1	265.1	64.0	76.3	116.3
**Ho**	8.1	6.2	2.2	1.1	0.7	0.8	0.1	0.4	6.1	9.7	2.9	2.8	4.5
**Er**	23.0	18.0	6.3	3.3	2.0	2.3	0.5	1.2	19.6	30.3	7.6	7.2	11.3
**Tm**	3.4	2.7	0.9	0.5	0.3	0.3	0.1	0.2	2.9	4.4	1.0	0.9	1.3
**Yb**	22.6	17.6	6.2	3.4	2.2	2.3	0.5	1.4	19.0	27.6	5.6	4.5	7.1
**Lu**	3.1	2.4	0.8	0.5	0.3	0.3	0.1	0.2	3.0	4.3	0.6	0.5	0.8
**TREE**	1001	657	239	158	110	111	111	288	1964	1543	738	484	634
**LREE%**	63	56	57	66	71	67	94	94	85	71	83	73	68
**HREE%**	37	44	43	34	29	33	6	6	15	29	17	27	32
**LREE (ppm)**	645	376	140	106	78	76	104	271	1680	1104	616	355	435
**HREE (ppm)**	367	287	101	53	32	36	7	18	289	444	125	131	203

Supplementary Table 2

Rare Earth Element concentrations at each step in the Imerys Kaolinite leaching and re-adsorption experiments. Measured after Lithium metaborate fusion/nitric acid digestion.

**Kaolinite/Standard**	**La**	**Ce**	**Pr**	**Nd**	**Sm**	**Eu**	**Gd**	**Tb**	**Dy**	**Y**	**Ho**	**Er**	**Tm**	**Yb**	**Lu**
	in mg kg^{-1} (ppm)														
**Solution Blank**	0.23	0.24	0.24	0.22	0.28	0.27	0.24	0.25	0.23	0.24	0.24	0.23	0.24	0.25	0.24
**1) Imerys Kaolinite**	12.63	28.90	3.54	13.18	3.25	0.51	3.08	0.50	2.55	13.15	0.47	1.18	0.21	1.13	0.17
Pre-Leach	12.56	29.03	3.52	12.98	3.26	0.47	2.94	0.46	2.56	12.73	0.40	1.10	0.17	1.10	0.14
**2) Imerys Kaolinite**	11.70	26.96	3.24	12.72	3.17	0.40	2.81	0.44	2.39	10.87	0.39	1.01	0.15	1.05	0.15
Post-leaching in $(NH_4)_2SO_4$	11.24	25.48	3.09	11.66	2.89	0.39	2.74	0.43	2.29	10.57	0.37	0.99	0.15	1.01	0.14
	9.98	23.17	2.72	10.48	2.50	0.35	2.38	0.37	2.00	9.12	0.33	0.86	0.13	0.87	0.11
**3) Imerys Kaolinite**	10.91	23.82	5.19	12.31	5.65	4.34	6.02	4.55	6.11	12.39	4.47	5.04	4.73	6.10	5.33
Re-adsorbed in $REENO_3$	10.68	23.44	5.25	12.35	5.85	4.36	5.76	4.65	6.19	11.84	4.66	5.12	4.85	6.05	5.46
	11.25	24.42	5.18	12.97	5.96	4.22	5.97	4.62	6.59	12.11	4.59	5.14	4.84	6.06	5.49
**4) Imerys Kaolinite**	11.62	26.91	3.57	12.54	3.60	1.19	3.47	1.27	3.19	10.60	1.26	1.89	1.33	2.55	1.65
Re-adsorbed, $(NH_4)_2SO_4$ leached	10.50	23.99	3.15	11.16	3.12	0.79	2.91	0.86	2.47	9.83	0.82	1.48	0.91	2.08	1.32
**5) Imerys Kaolinite**	10.24	22.38	4.99	12.11	5.96	4.39	5.91	4.71	6.35	10.98	4.67	5.21	4.95	6.25	5.55
Re-adsorbed, H_2O leached	11.83	26.02	5.43	13.67	6.40	4.44	6.47	4.72	6.49	11.63	4.72	5.33	4.94	6.41	5.39
**BLANK_LiBO2**	BD	BD	BD	BD	BD	BD	BD	BD	BD	BD	BD	BD	BD	BD	BD
**Silica Blank_BLOI-3**	9.07	17.92	1.94	6.91	1.18	0.11	0.91	0.13	0.78	3.46	0.14	0.43	0.07	0.47	0.06
**CRM-BCR701**	56.77	122.60	13.74	51.84	10.15	1.77	9.69	1.40	8.38	37.80	1.72	4.92	0.70	4.74	0.63
**CRM-BCR-2**	26.36	56.87	7.15	29.67	6.65	2.19	7.54	1.13	6.89	30.20	1.44	4.31	0.60	3.83	0.54
**CRM-SBC-1**	61.48	133.01	15.14	58.37	11.83	2.39	10.96	1.49	8.66	34.91	1.78	5.01	0.70	4.66	0.68
**CRM-GSP-2**	204.77	493.36	61.02	224.16	28.11	2.53	15.18	1.47	6.39	22.79	1.09	2.78	0.32	1.98	0.25

Supplementary References

1. Estrade G, Marquis E, Smith M, Goodenough K, Nason P. REE concentration processes in ion adsorption deposits: Evidence from the Ambohimirahavavy alkaline complex in Madagascar. *Ore Geol Rev* **112**, 103027 (2019).

2. Xu C*, et al.* Origin of heavy rare earth mineralization in South China. *Nature Communications* **8**, 14598 (2017).

3. Bao Z, Zhao Z. Geochemistry of mineralization with exchangeable REY in the weathering crusts of granitic rocks in South China. *Ore Geol Rev* **33**, 519-535 (2008).

4. McDonough WF, Sun S-S. The composition of the Earth. *Chem Geol* **120**, 223-253 (1995).
