# Supplementary material for: Adsorption of rare earth elements in regolith-hosted clay deposits
Source: Nat Commun. 2020 Sep 1;11:4386. doi: 10.1038/s41467-020-17801-5 (PMC7463018; doi:10.1038/s41467-020-17801-5)
Supplement: Supplementary file 3 — Description of Additional Supplementary Files [file 41467_2020_17801_MOESM3_ESM.pdf]

Description of Additional Supplementary Files

Supplementary Data 1: Materials (Excel)

1.1 Samples
1.2 Prepared Clay standards
1.3 Mineral and solution standards

Explanation
Information on the samples and standards used in the study; (1.1) overview of the studied regolith-samples from China and Madagascar selected for X-ray Absorption Spectroscopy analyses, (1.2) materials used for the leaching and re-adsorption experiments of synthetic kaolinite and (1.3) the mineral standards containing REE in various coordination states, measured as standards during the X-ray Absorption Spectroscopy analyses

Supplementary Data 2: Y XANES (Excel)

2.1 Normalised Y XANES data for sample LJ316, China
2.2 Normalised Y XANES data for PIT2, Madagascar
2.3 Normalised Y XANES data for samples MAD238 and MAD217, Madagscar
2.4 Normalised Y XANES data for prepared clay samples
2.5 Un-normalised Y XANES data for prepared clay samples
2.6 Normalised Y XANES data for standards

Explanation
Y K-edge XANES spectra for samples and standards. Measurements carried out at the I18 beamline of Diamond Light Source. Absorption spectra are plotted as a function of energy (eV) and were processed in Athena. Details on the experimental set-up at I18, and of the normalising procedures in Athena are provided in the paper. Athena Source Files provided in Supplementary Data 5.

Supplementary Data 3: Nd XANES (Excel)

3.1 Normalised Nd L3-edge XANES of sample LJ316, China
3.2 Normalised Nd L3-edge XANES of PIT2 and MAD217, Madagascar
3.3 Normalised Nd L3-edge XANES of standards
3.4 Normalised Nd L3-edge XANES of prepared clay (Imerys kaolinite) standards

Explanation
Normalised Nd L3-edge X-ray Absorption Near Edge Structure (XANES) spectra for minerals and standards. Measurements carried out at the I18 beamline of Diamond Light Source. Normalised absorption spectra (.nor) are plotted as a function of energy (eV) and were processed in Athena. Details on the experimental set-up at I18 and normalising procedures in Athena are provided in the paper. Athena Source Files provided in Supplementary Data 5.

Supplementary Data 4: Y EXAFS data and fits (Excel)

4.1 Y EXAFS Data and Fits for clay-hosted Y in samples
4.2 Y EXAFS Data and Fits for mineral standards

Explanation

K2-weighted EXAFS data and best fits obtained in ARTEMIS. EXAFS functions are plotted as a function of wavenumber k (Å-1). Fourier Transform Radial Distribution functions (R and Rreal) are plotted as a function of Radial Distance R (Å). Details of fitting procedure and structural models used for the mineral standards are provided in the paper.

ARTEMIS source files of EXAFS fitting are provided in Supplementary Data 6.

Supplementary Data 5: ATHENA .txt files (folder)

5.1 Y XANES data

5.2 Nd XANES data

Explanation

Athena Source files for the XANES data presented in the paper

Supplementary Data 6: ARTEMIS .prj files (folder)

ARTEMIS Source files for the EXAFS fits provided in Supplementary Data 4
